# Sustainable thresholds, health outcomes, health expenditures and education nexus in selected African countries: quadratic and moderation modelling

**DOI:** 10.1186/s12992-022-00876-8

**Published:** 2022-10-12

**Authors:** Yetunde Oluranti Adegoke, Gavin George, Josue Mbonigaba

**Affiliations:** 1 School of Accounting, Economics and Finance, University of KwaZuluNatal, Durban, South Africa; 2 Health Economics and HIV and AIDS Research Division (HEARDS), University of KwaZulu-Natal, West-Ville Campus, Durban, South Africa; 3 School of Accounting, Economics and Finance, University of KwaZulu-Natal, Durban, South Africa

**Keywords:** Maternal mortality, Child mortality, Life expectancy, health expenditures, Education, Quadratic modelling, Moderation modelling, Africa, PSCC, I19, C24

## Abstract

**Background:**

This study aligns with Sustainable Development Goal 3 which borders on “*good health and well-being for people by ensuring healthy lives and promoting well-being for all at all ages”*. It contributes to the health literature by evaluating the roles of health expenditures and educational quality on three health outcomes (infant mortality, maternal mortality and life expectancy at birth).

**Methods:**

The study uses the panel spatial correlation consistent (PSCC) approach on balanced panel data on 25 selected sub-Saharan African countries from 2000 to 2020 to interrogate the nexus.

**Results:**

The following findings are documented. First, health expenditures reveal significant asymmetric quadratic effects on health outcomes. Second, the interactions between health expenditures and educational quality reduce infant and maternal mortalities while enhancing life expectancy. Third, the threshold points from the interaction effects indicate that enhancing educational quality beyond some critical thresholds of 1.51 and 1.49 can induce a drop in maternal and child mortalities while a point beyond 1.84 exerts an improvement in life expectancy.

**Conclusions:**

Hence, policy makers should ensure that both health expenditures and educational quality exceed the established thresholds for sustainable health outcomes.

## Introduction

Universal discussions on health issues and challenges have been centred on the need for increased government funding for health care as a result of the growing international focus on the need for adequate government spending on a range of social services, including health care especially in sub-Saharan Africa (SSA). Where public expenditures on healthcare services are pervasively poor. Public health expenditure is an indispensable prerequisite for effective and efficient health sector performance. Health expenditure is important because it provides resources and economic incentives for the operation of the health systems; and it is the key determinant of health sector performance in terms of equity, efficiency and health outcomes [17]. Likewise, educational quality plays an important role in the delivery of better health outcomes [29]. In sub-Saharan African the health outcomes are generally poor i.e., high maternal and child mortalities, consequently, the life expectancy at birth is low when compared with other regions of the world. The life expectancy at birth (LEB) was averagely 62 in 2019 for SSA, while, in the low-income countries, the average LEB was 64. Middle income countries, 72 and 81 in high income countries. Moreover, the United Nations Millennium Development Goals (MDGs) from 2000 to 2015 and Sustainable Development Goals (SDGs) from 2015 to 2030 have underscored the importance of good health for economic development. In the MDGs, three out of eight goals were, dedicated to health improvement of people [26, 27]. These are to, reduce child mortality; improve maternal health; combat human immunodeficiency virus (HIV), acquired immune deficiency syndrome (AIDS), malaria and other diseases. Similarly, the Goal 3 of SDGs is centred on good health and well-being for people by ensuring healthy lives and promoting well-being for all at all ages. The performance of most African countries, especially sub-Saharan African countries was very far below par as far as MDGs and SDGs are concerned, by and large, the introduction of these goals has revealed the importance of healthcare expenditure and financing in the region. Over the years, SSA countries have made significant efforts in increasing health expenditures, with the aim of improving health outcomes. Despite the increase in current health expenditures, health outcomes responded marginally [6]. Probably, because the existing health frameworks failed to account for the accurate mix of health expenditure and educational quality that could engender improved health outcomes. Consequentially, leading to a gap in this regard. The focus of the extant literature has been on the effect of health expenditure (public or private) on health outcomes without much emphasis on the moderating effect of education quality and health expenditure on health outcomes.

**Fig. 1:**
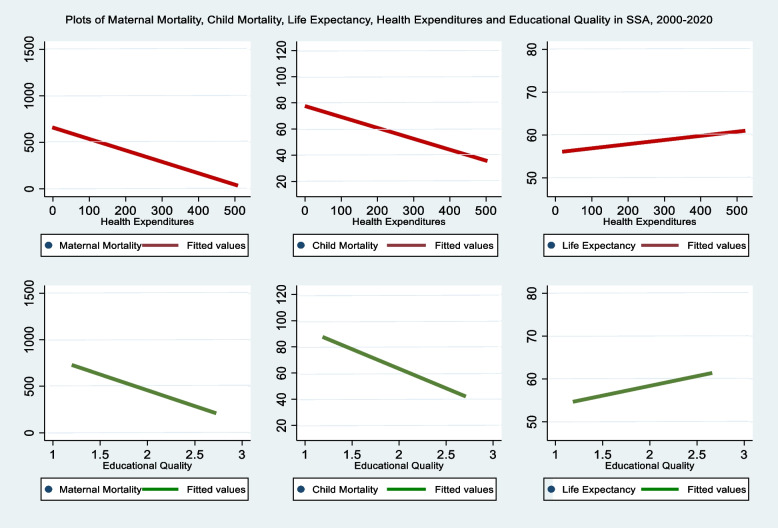
Note- Ang-Angola, Bur- Burundi, Ben- Benin, B.fso- Burkina Faso, Bots- Botswana, Car- Central Africa Republic, Cot- Cote d’ Ivoire, Cam- Cameroon, Esw- Eswatini, Gam- Gambia, Ken- Kenya, Les- Lesotho, Moz- Mozambique, Maur- Mauritian, Mau- Mauritius, Nam- Namibia, Niger- Niger, Nig-Nigeria, Rwa- Rwanda, Sud-Sudan, Sen- Senegal, Sleo- Sierria leone, S.A- South Africa, Tanz- Tanzania, Togo- Togo

As expected, if government and institutional stakeholders allocate resources to the health and educational sectors in addition to permitting an enabling environment, an enabling environment can be ensured through a timely intervention of government in controlling corruption, inflation and other economic problems. Therefore, it is rational to find a reduction in maternal and child deaths while the lifespan of the population is enhanced, ceteris paribus*.* Figure 1 shows the scatterplots evidencing these intrinsic relationships. We find that maternal and child mortalities reveal a negative association with health expenditures and educational quality while a positive association is evident with life expectancy. These outcomes are unsurprising and documented in the literature.

Drawing from Fig. 1, this study focuses on three health outcomes: maternal mortality, child mortality, and life expectancy at birth. The examination of the “health expenditure”, “educational quality” and “sustainable thresholds” hypotheses is motivated by the relatively poor performance of health outcomes in Africa and the apparent gap in the extant literature. Extending the work of Barua et al. [7], this study’s objectives are to: (i) determine the health expenditure thresholds on health outcomes; (ii) assess if educational quality improves or distorts the effect of health expenditures on health outcomes; (iii) evaluate the conditional effect of health expenditures on health outcomes; and (iv) provide stakeholders with minimum thresholds of educational quality required to sustain health expenditures in influencing health outcomes. Pooled and fixed effects estimation techniques are engaged to methodically probe these questions with an unbalanced panel data of ten variables on 25 selected Sub-Saharan African countries. The rest of the study is structured as follows: Literature review section discusses the empirical literature; Methodology section outlines the data and model; Results and discussion section interprets the results and Conclusion and policy implications section concludes with policy recommendations.

## Literature review

The nexus between health expenditures and health outcomes is well established in the literature for both developing and developed countries. For example, Onofrei, Vatamanu, Vintila and Cigu [22], analysed the relationship between public health expenditure and health outcomes among European Union developing countries via regression and factor analysis. The study concluded that public health expenditure can improve life expectancy at birth and infant mortality. Ibukun [17], examined the mediating role of governance in health expenditure-health outcomes nexus for West African countries via two stage least square estimating technique for a panel of 15 countries from 2000 to 2018. They adopted three indices of health outcomes and six indices of governance. The result showed that all types of health expenditure significantly affect all health outcomes namely infant mortality, under –five mortality and life expectancy at birth. Rahman, Khanam and Rahman [24] examined the health care expenditure and health outcomes nexus for South Asian Association for Regional Cooperation-Association of South East Asian Nations (SAARC-ASEAN). Three types of health expenditure namely, private, public and total health expenditure were incorporated and three main health status outcomes namely, life expectancy at birth, crude death rate and infant mortality rate were used. The study involved 15 countries and spanned from 1995 to 2014. The data was analysed via the Fixed and random effects model. The study reported that total health expenditure, public health expenditure and private health expenditure significantly reduced infant mortality rates. While, the extent of the effect of private health expenditure was higher than the public health expenditure. The latter also had a significant effect in reducing crude death rates.

Rana, Alan and Gow [25] investigated the relationship between health expenditure and health outcomes for 161 developed and developing countries with different income levels for a period of 1995 to 2014. This study adopted a panel autoregressive distributed lag (PARDL). The result of the study revealed that the relationship between health expenditure and health outcome is stronger for low-income countries compared to high income countries. The study also reported that variations in child mortality are better explained by increase in health expenditure than in maternal mortality in all income levels. The study concluded that health expenditure had no significant effect on maternal mortality. The income level might have better impact in low-income countries because the epidemiological profile in the low-income countries is higher than the high-income countries, therefore, a small increase in health expenditure will surely have high impact on the high mortality and morbidity in the low-income countries than it would have had in the high-income countries.

Munteh and Fonchin [20] investigated the impact of public health expenditure on under five mortality in Cameroon, ordinary least square regression was employed to analyse the data. The results revealed that public health expenditure had a negative but insignificant effect on under five mortality. Njoroge [21] investigated the impact of health expenditure on health outcome in 18 East and Southern Africa countries with emphasis on the role of governance. The study period spans from 2001 to 2017, the study employed generalized method of moments (GMM) as the estimating technique. The results revealed that health expenditure (total, public and private) had significant negative relationship with all the health outcomes (under five mortality and maternal mortality) while positive relationship was documented between health expenditure and life expectancy at birth.

Kilanko [18] investigated the impact of health expenditure on infant mortality and under five mortality among the West Africa countries from 2000 to 2018. Fixed and Random effect was employed to analyse the data, the results revealed a significant negative relationship with all the health outcomes considered. Danladi [12] investigated the impact of health expenditure on life expectancy at birth in Nigeria. The data spans from 1979 to 2019, Dynamic ordinary least square was employed. The results revealed an insignificant positive relationship between health expenditure and life expectancy at birth.

Henock [15] investigated the impact of government health expenditure on health outcome in Southern Africa countries from 2000 to 2016. Fixed effects regression was employed for the analyses the results of the findings revealed the significant impact of public health expenditure on the mortality outcomes but insignificant in regards to life expectancy at birth. Amposah [4] investigated the impact of health expenditure and other macro-economic determinants on health outcome in SSA countries. The data spans from 2000 to 2015. The results revealed GDP per capita, physician per 1000 population, population aged above 65 years and under five mortalities as determinants of health expenditure. While, health expenditure had a significant negative impact on maternal and infant mortality but a positive significant impact was recorded in regards to life expectancy at birth.

Bein, Unlucan and Olowu [8] investigated the impact health expenditure on health outcome proxied as infant mortality and life expectancy at birth among 10 Eastern countries. Fixed effects regression analysis was employed in the study. The results revealed a positive relationship between health expenditure and life expectancy at birth. While a negative relationship was recorded between health expenditure and health outcome proxied by neonatal, infant and under five mortalities.

Similarly, the nexus between education and health has been considered in the literature, see the work of Xue [29], in the study a meta-analysis method for 105 studies with 4671 estimates was employed to explain the education-health nexus. The study finds education as a significant determinant of health outcomes. While, the education-health nexus is positive in most of the studies considered, Xue, concluded that most of the studies that do not control for endogeneity are prone to exaggerating the estimated effect but the effect becomes weaker in more recent studies. Raghupathi and Raghupathi [23] investigated the influence of education on health for Organisation for Economic Co-operation and Development (OECD) countries from 1995 to 2015, health data from 26 OECD and World Bank was used. The variables included in the study were education proxied by enrolment rates at various educational levels, NEET (Not in employment, education or training) rate, school life expectancy, while, the health indicators are proxied by infant mortality. Child vaccination rates, deaths from cancer, life expectancy at birth, potential years lost and smoking rates, the data was analysed with tools of tableau for visualization and Statistical Analytics Software for correlation and descriptive statistics. Adults with higher educational attainment have better health and life span compared to their less-educated peers. The study found tertiary education as critical in influencing infant mortality, life expectancy and child vaccination. Despite, the escalation of studies on health expenditure-health outcomes nexus and education-health outcomes nexus the modifying effect of education and health expenditure on health outcomes is missing in the literature. Therefore, this study examines the modifying effect of education and health expenditure on health outcomes and also estimate minimum thresholds of public health expenditure and educational quality necessary for better health outcomes.

## Methodology

### Estimation techniques

For the main analysis, we use the panel spatial correlation consistent (PSCC) pooled ordinary least squares (PSCC-OLS) approach and for robustness checks control for fixed effects using the PSCC-FE methods. These techniques are suitable in the event of cross-sectional dependence in the data and have been used in various panel data studies [1, 2]. The PSCC estimator uses the Driscoll and Kraay [13] robust standard errors technique and corrects the standard errors of the coefficient estimates for possible dependence [11, 16]. The underlying algorithm routines the OLS/WLS^1^^,^^2^ (Ordinary Least Square/ Weighted Least Square) and fixed effects (within) regression and computes spatial correlation consistent standard errors for linear panel models. Contextually, cross-sectional dependence can be defined as: “some correlation structure in the error term between [cross-sectional] units” [10]. Hence, the test for cross-sectional dependence (CSD) is to “test whether the residuals are correlated across entities”. The null hypothesis is “there is no correlation of the residual”. There may be the possibility of CSD in the data if for instance, a Nigerian migrant who lives in South Africa has access to maternal and child care. In the event that the Nigerian relocates back to her country such maternal and child care becomes inaccessible which may cause some health challenges. Also, reverse causality might be a concern. For instance, health expenditure is likely to be larger in countries with greater health issues and therefore higher mortality. To control this, the models are estimated using one-period lag of each regressor thereby mitigating the problem of reverse causality. Given this, we are confident that the obtained results are unbiased and somewhat precise for inferences. Next, the study deploys the fixed effects approach (PSCC-FE) which serves as robustness checks accounts for heterogeneities across the panel. For additional robustness checks, we used (1) people living with tuberculosis in place of those living with HIV to observe if our results holds or consistent; (2) 5-year averages for both HIV and tuberculosis (TUB) models to observe if the initial results are sustained.

### Variables and expectations

The study uses a total of ten variables on 25 SSA countries^2^ from 2000 to 2020 to probe the study questions and achieve the objectives. There are three dependent variables representing the health outcomes: maternal mortality (MAT), child mortality (CHD) and life expectancy at birth (LEX). The main explanatory variable is health expenditures per capita (HEXPC), the moderating variable is human capital index as the proxy for education (EDU), the control variables are: Internet users (ICT), household consumption (HCON), individuals living with human immunodeficiency virus (HIV), people with tuberculosis (TUB) and total factor productivity (TFP). All the health variables were sourced from World Development Indicators [28] while, the non-health variables were sourced from Penn World Table (PWT 9.0).

On a priori expectations, we expect negative relationship between public health expenditure and health outcomes such that higher health expenditure may be expected to improve health outcomes by reducing maternal and child morbidity/mortality [3, 5, 17, 24]. Also, negative relationship is expected between the health outcomes and public health expenditure per capita, ICT, household consumption and total factor productivity. In the contrary, positive relationship is expected between the health outcomes i.e. maternal and child mortality and other health variables such as human immunodeficiency virus (HIV) and people living with tuberculosis, given argument traceable to Moran and Moodley [19]. While, between life expectancy at birth and public health expenditure a positive relationship is expected [22]. The same direction of relationship (positive) is also expected between education and life expectancy at birth (as well as ICTs as contemporary tools for education and other variables such as total factor productivity, household consumption).

### Model

In determining health expenditure thresholds which addresses the first objective, each health outcome is expressed as a function of health expenditures, a quadratic specification, educational quality, and a set of control variables. That is:

1 $$\ln {HO}_{it}={\eta}_0+{\eta}_1\ln {HEXPC}_{it}+{\eta}_2\ln {HEXPC}_{it}^2+{\eta}_3\ln {EDU}_{it}+{\theta}^{\prime }{\boldsymbol{Z}}_{it}+{\phi}_t+{v}_{it}$$

Where, ln is natural logarithm; *HO* represents each health outcome (maternal mortality, child mortality, life expectancy at birth); *HEXPC* is health expenditures per capita; *EDU* is educational quality; ***Z*** is a vector of control variables (ICT, HCON, HIV, TFP); *ϕ*_*t*_ represents year dummies; *η*_*i*_ and *θ*^′^ are parameters to be estimated; and *v* is the idiosyncratic error term assumed to be white noise. To control for outliers, establish an elasticity relationship and reduce “noise” in the data, all variables are transformed into their natural logarithms.

To determine the health expenditures threshold, Eq. (1) assumes homogeneity for the parameters *η*_1_, and *η*_2_ which depend neither on a specific country nor on the time period. It is assumed that all countries take on the same shape of the functional relation of the health outcomes-expenditures paradox. More importantly, Eq. (1) allows for testing the various forms of the relationships viz.: (i) *η*_1_ < 0, *η*_2_ > 0 reveals a U-shaped relationship; (ii) *η*_1_ > 0, *η*_2_ < 0 reveals an inverse U-shaped relationship. The health expenditures turning point of this curve is computed by $\hat{\tau}=\exp \left(0.5 {\hat{\eta}_1}\!\left/ \ {\hat{\eta}_2}\right.\right)$; (iii) *η*_1_ > 0, *η*_2_ > 0 reveals a monotonically increasing linear relationship; (vi) *η*_1_ < 0, *η*_2_ < 0 reveals a monotonically decreasing linear relationship; and (vii) *η*_1_ = 0, *η*_2_ = 0 reveals a level relationship. In general, the turning point is when the first derivative of Eq. (1) with respect to health outcomes is equated to zero.

To achieve the second, third, and fourth objectives, each health outcome is expressed as a function of health expenditures, educational quality, an interaction term of health expenditures and educational quality, (ln*HEXPC*_*it*_ ∗ ln *EDU*_*it*_), and a set of control variables. That is:

2 $$\ln {HO}_{it}={\phi}_0+{\phi}_1\ln {HEXPC}_{it}+{\phi}_2\ln {EDU}_{it}+{\phi}_3\left(\ln {HEXPC}_{it}\ast \ln {EDU}_{it}\right)+{\psi}^{\prime }{\boldsymbol{K}}_{it}+{\omega}_t+{s}_{it}$$

Adapting Brambor et al. [9] and Barua et al. [7], the sign of the coefficient of the interaction term, *ϕ*_3_ evaluates if the interaction of *EDU* and *HEXPC* enhances or distorts the impact of health expenditures on health outcomes. Since *ϕ*_1_ is expected to be negative for mortality models and positive for life expectancy model which is a “good effect”, a positive (negative) *ϕ*_3_ indicates that *EDU* distorts (improves) the “good effect” of health expenditures on maternal and child mortalities while it enhances (reduces) the “good effect” of health expenditures on life expectancy at birth. Therefore, the conditional effect of *HEXPC* on health outcomes is computed as:

3 $$\frac{\partial \ln HO}{\partial \ln HEXPC}={\phi}_1+{\phi}_3\ln EDU$$

From Eq. (3), the overall effect of *HEXPC* on health outcomes depends on the estimated signs of *ϕ*_1_ and *ϕ*_3_, their respective statistical significance and the magnitude of *EDU*. But if either *ϕ*_1_ and *ϕ*_3_ = 0, then the conditional effect cannot be evaluated. Our prior is that increasing health expenditures will reduce mortality rates but increase life expectancy in countries exhibiting a high degree of educational quality. Such that *ϕ*_3_ < 0 is expected for mortality models and *ϕ*_3_ > 0 for life expectancy model. Thus, the minimum thresholds of educational quality required to sustain health expenditures in influencing health outcomes is derived as: $\ln {EDU}^{\ast }=-\frac{\phi_1}{\phi_3}$. Where, ln*EDU*^∗^ denotes the threshold of educational quality beyond which health expenditures reduce maternal and child mortality rates but increase life expectancy at birth.

## Results and discussion

### Pre-estimation results

Table 1 contains the descriptive statistics. Furthermore, it displays the correlation coefficients between the target variables (lnMAT, lnCHD and lnLEX) and the regressor variables (lnHEXPC, lnEDUC, lnICT, lnHCON, lnHIV, lnTUB and lnTFP). The closer the correlation coefficient is to − 1 or 1, the stronger the association [14]. It is critical to indicate that while the correlation matrix may measure the direction and strength of association between dependent and independent variables, this does not always indicate causality. On the whole, all variables of interest are significant at 1% level as shown by the estimated correlation coefficients. The strength of the relationship in most cases is quite strong and have the expected signs with no indication of multicollinearity among the regressors. Also, the cross-sectional dependence test shows (last column of Table 1) that there is no cross-sectional independence as the coefficient are all significant.

**Table 1:** Summary statistics, pairwise correlations and cross-sectional dependence results

Variable	Summary Statistics					Pairwise Correlations			CSD-Test
	Obs	Mean	Std. Dev.	Min	Max	lnMAT	lnCHD	lnLEX	
LnMAT	525	550.133	332.249	52	2480	1.000			64.870***
LnCHD	525	71.095	26.195	13.262	147.185	0.827***	1.000		37.693***
LnLEX	525	56.982	7.049	39.441	74.515	−0.677***	− 0.744***	1.000	74.414***
lnHEXPC	525	110.342	161.28	3.395	844	−0.694***	−0.533***	0.322***	34.962***
LnEDU	525	1.753	0.449	1.069	2.939	−0.586***	−0.563***	0.343***	65.981***
LnICT	525	11.427	13.902	0.026	59.42	−0.602***	−0.566***	0.617***	75.210***
LnHCON	525	0.709	0.151	0.221	1.039	0.326***	0.076*	−0.191***	1.734*
LnHIV	525	6.459	7.939	0.2	28.9	−0.153***	0.021	−0.369***	17.337***
LnTUB	525	363.395	325.685	11	1590	0.267***	0.381***	−0.567***	37.413***
LnTFP	525	0.472	0.227	0.142	1.25	−0.551***	−0.491***	0.223***	6.021***

Notes: *** *p*<0.01, ** *p*<0.05, * *p*<0.1, t-statistics in parentheses, *ln* Natural logarithm, *MAT* Maternal mortality, *CHD* Child mortality, *LEX* Life expectancy at birth, *HEXPC* Health expenditures per capita, *EDUC* Education, *ICT* Internet users, *HCON* Household consumption, *HIV* People living with HIV, *TFP* Total factor productivity

Source: Authors’ Calculations

### Composite main results, PSCC-OLS and PSCC-FE

Table 2 is a composite result showing the quadratic results (Eq. 1) and moderation results (Eqs. 2 and 3) across the three dependent variables: maternal mortality (columns 1,4,7,10), child mortality (columns 2,5,8,11), and life expectancy at birth (columns 3,6,9,12) and two estimation techniques. Results are interpreted sequentially along modelling structure.

**Table 2:** Composite main results (HIV models)

Variables	PSCC-OLS						PSCC-FE					
	Quadratic Models			Moderation Models			Quadratic Models			Moderation Models		
	lnMAT [1]	LnCHD [2]	lnLEX [3]	lnMAT [4]	lnCHD [5]	lnLEX [6]	lnMAT [7]	lnCHD [8]	lnLEX [9]	lnMAT [10]	lnCHD [11]	lnLEX [12]
LnHEXPC	0.708*** (8.702)	0.175*** (3.084)	−0.0594** (− 2.312)	0.307*** (7.641)	0.126*** (8.440)	− 0.0604*** (− 5.219)	− 0.280*** (− 6.173)	−0.172*** (− 3.447)	0.0350** (2.133)	− 0.162*** (− 5.947)	−0.0914** (− 2.432)	0.00148 (0.116)
LnEDU	0.189** (2.359)	−0.579*** (− 12.86)	0.109*** (7.275)	2.938*** (18.62)	0.626*** (5.407)	−0.282*** (−6.116)	− 0.463*** (− 5.533)	−0.842*** (− 10.28)	0.0569** (2.631)	− 1.787*** (− 7.528)	− 1.288*** (− 5.522)	0.0315 (0.280)
LnICT	− 0.298*** (− 10.92)	− 0.164*** (− 7.747)	0.0371*** (6.771)	− 0.357*** (− 10.82)	−0.199*** (− 9.444)	0.0494*** (8.728)	− 0.0702*** (− 7.849)	−0.00831 (− 0.956)	0.00898*** (3.234)	− 0.0483*** (− 5.963)	− 0.00317 (− 0.270)	0.0114** (2.450)
LnHCON	0.0937** (2.308)	− 0.425*** (− 12.78)	− 0.0169** (− 2.472)	0.0237 (0.619)	− 0.456*** (− 12.29)	−0.00660 (− 1.123)	−0.0220 (− 0.380)	−0.0324 (− 1.122)	0.00852 (0.785)	− 0.0400 (− 0.649)	−0.0354 (− 1.169)	0.00540 (0.495)
LnHIV	0.0998*** (11.57)	0.131*** (19.45)	−0.0534*** (− 10.95)	0.0739*** (7.747)	0.127*** (20.69)	−0.0528*** (− 11.56)	− 0.166*** (− 5.990)	0.00154 (0.208)	0.0179 (1.208)	− 0.173*** (− 5.560)	0.00952 (1.523)	0.00862 (0.640)
LnTFP	− 0.0707 (− 0.915)	−0.0406 (− 1.632)	−0.00554 (− 0.395)	−0.0188 (− 0.327)	−0.0108 (− 0.499)	−0.0161 (− 1.334)	0.0579 (1.257)	− 0.0165 (− 1.113)	−0.0130 (− 0.846)	0.0565 (1.257)	−0.0228 (− 1.665)	−0.00790 (− 0.519)
LnHEXPCSQ	− 0.112*** (− 13.59)	−0.0321*** (− 5.304)	0.00814*** (3.198)				0.0405*** (6.207)	0.0197*** (3.643)	−0.00455* (− 1.830)			
lnHEXPC*lnEDU				−0.737*** (− 20.84)	− 0.310*** (− 16.13)	0.0988*** (9.237)				0.280*** (7.285)	0.0933** (2.560)	0.00621 (0.299)
**Threshold**	**3.16**	**2.73**	**3.65**	**0.42**	**0.41**	**0.61**	**3.46**	**4.37**	**3.85**	**0.58**	**0.98**	**NA**
Constant	0	0	0	0	0	0	0	0	0	0	0	0
Year Dummies	Yes	Yes	Yes	Yes	Yes	Yes	Yes	Yes	Yes	Yes	Yes	Yes
Observations	525	525	525	525	525	525	525	525	525	525	525	525
R-squared	0.644	0.584	0.643	0.679	0.610	0.683						
Countries	25	25	25	25	25	25	25	25	25	25	25	25
F-Statistic	5201	10,942	7154	771.5	1312	812.7	784.2	1244	665.7	58.55	199.2	35.90

Source: Authors’ Calculations

*ln* Natural logarithm, *MAT* Maternal mortality, *CHD* Child mortality, *LEX* Life expectancy at birth, *HEXPC* Health expenditures per capita, *EDUC* Education, *ICT* Internet users, *HCON* Household consumption, *HIV* People living with HIV, *TFP* Total factor productivity

*** *p* < 0.01, ** *p* < 0.05, * *p* < 0.1; *t*-statistics in parentheses

For the quadratic models, we are only interested in the coefficients of HEXPC and HEXPCSQ to observe the shape of the curve. For maternal and child mortality, the relationship with health expenditures evidenced an inverted U-shape curve. That is, initial increase in health expenditures causes a rise in both maternal and child mortality. However further increase in health expenditures results in a decline in mortalities supporting the literature stance on the mortality-reducing effect of health expenditures [17]. In the same vein, life expectancy and health indicate a U-shape relationship. That is, initial increase in health expenditures causes a decline in life expectancy but additional increase in health expenditures results in improving life expectancy Again, this finding aligns with the empirical literature [22].

**Fig. 2:**
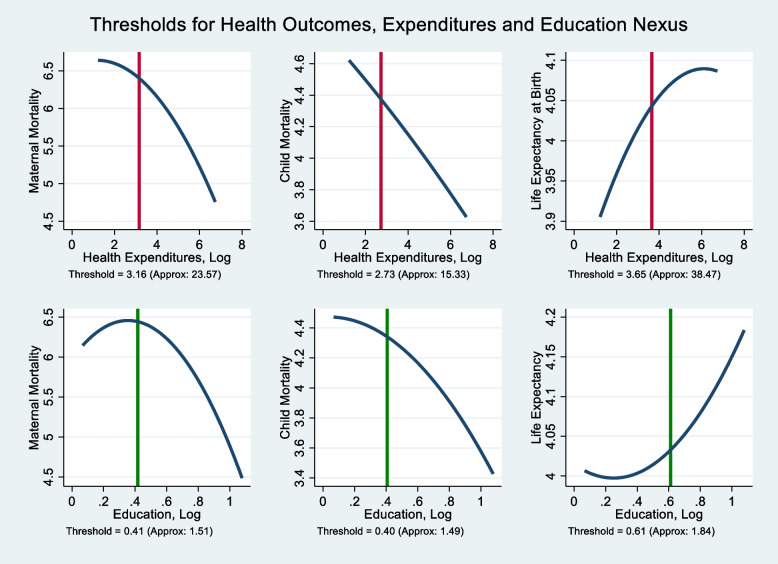
Sustainable thresholds of health expenditures and educational quality. Source: Authors’ Computations

From the quadratic results, we obtain the sustainable thresholds or turning points beyond which health expenditures exert significant impact on health outcomes. From Eq. 1, the health expenditures turning point for maternal mortality is computed as $\hat{HEXPC}=0.5\ast {}^{0.708}\!\left/ \!{}_{0.112}\right.$ = 3.16; for child mortality: $0.5\ast {}^{0.175}\!\left/ \!{}_{0.0321}\right.$ = 2.73, and life expectancy: $0.5\ast {}^{0.0594}\!\left/ \!{}_{0.00814}\right.$ = 3.65. Since, these equations are computed using natural logarithm, it becomes important to take the exponents so as to confirm if these thresholds lie within the range of data. Therefore, for each health outcome, the corresponding sustainable health expenditures thresholds in real terms are as follows: maternal mortality: [exp(3.16)] = 23.57, child mortality: [exp(2.73)] = 15.33, and life expectancy: [exp(3.65)]= 38.47. Recall that from Table 1, the range of values for health expenditures per capita is 3.395 to 844 and since the shape for the parabola for the mortality models is an inverted U-shape suggesting that beyond 23.57 and 15.33, health expenditures per capita will contribute significantly to reducing both maternal and child mortality. In the same vein, following the U-shape relationship, it follows that beyond the threshold point of 38.47, health expenditures per capita will contribute significantly to improving life expectancy. We show this graphically in Fig. 2.

Having established the quadratic effects, this study proceeds with the results of the moderation models to compute the minimum sustainable thresholds at which educational quality could enhance the effect of health expenditures on each health outcome. These thresholds have policy implications because beyond the critical masses, the effect of health expenditures on each health outcome is dependent on the strength of educational quality. Given these clarifications, only the significant coefficients of HEXPC and HEXPC*EDUC are used in computing the threshold points. We observe an inverted U-shaped curve exists between health expenditures, educational quality and the mortality models while a U-shaped curve hold for life expectancy model. Following Eq. 4, the threshold points for ln*EDU*^∗^ across each health outcome are: Maternal Mortality: $-\frac{0.307}{-0.737}=0.42$; Child Mortality: $-\frac{0126}{-0.310}=0.41$; and Life Expectancy: $-\frac{-0.0604}{0.0988}=0.61$. In these computations, 0.307, 0.126 and − 0.0604 represent the absolute value of the unconditional effect of health expenditures on each health outcome while − 0.737, − 0.310, and 0.0988 represent the moderation/conditional effect between health expenditures and educational quality on each health outcome. Hence, from these computed thresholds, it holds that threshold points beyond 0.42 and 0.41 induce a drop in maternal and child mortalities while a point beyond 0.61 exerts an improvement in life expectancy.

Similar to the quadratic thresholds, those computed from the moderation models are done using the natural logarithm. Therefore, to ascertain that these points lie within the range of educational quality we take the exponents to obtain the corresponding values as: maternal mortality: [exp(0.42)] = 1.52, child mortality: [exp(0.41)] = 1.51, and life expectancy: [exp(0.61)] = 1.84. Also, from Table 1, the range of values for educational quality is 1.069 to 2.939 and since the shape for the parabola for the mortality models is an inverted U-shape signifying that beyond 1.52 and 1.51, educational quality improves the mortality-reducing potentials of health expenditures per capita on maternal and child mortality. Similarly, beyond 1.84, educational quality improves the life-enhancing effect of health expenditures per capita on life expectancy per capita. Also, these outcomes are shown in Fig. 2.

The PSCC-OLS does not recognise the individual heterogeneities or fixed effects across the countries in the panel, hence, we re-estimated the models using the fixed effects routine (PSCC-FE) and the results are shown in the second half of Table 2. For the most part, the obtained results are consistent with those of PSCC-OLS. Though the quadratic models reveal that health expenditures have a U-shaped relationship with maternal and child mortality; and an inverted U-shape curve with life expectancy suggesting that more allocations of health expenditures beyond an identified threshold worsens health outcomes and the respective calculated thresholds are 3.46, 4.37 and 3.85, respectively. Analogous to the calculations done in Table 2, the real values for these threshold points lie within the range of health expenditures (3.395 to 844) are: 32.14, 79.04, and 46.99. The most plausible argument for these contradictions could be that individual differences across the countries is driving these anomalies.

Lastly, the moderation models reveal a U-shaped curve between health expenditures, educational quality and the mortality models while the relationship with life expectancy is not different from zero (that is, statistically not significant). Computing the critical mass for sustainable resulted in 0.58 and 0.98 for maternal and child mortality, respectively which that of life expectancy is inconclusive.^3^ Converting to real terms, the threshold points which lie within the range of values for educational quality are 1.79 and 2.66 beyond which educational quality *negatively* influences the impact of health expenditures on maternal and child mortality.

### Robustness results

To test the robustness of our analysis, we engage two sets of sensitivity checks. The first uses people living with tuberculosis (TUB) in place of those with HIV. The second uses 5-year averages of the sample data with HIV and TUB models across both empirical techniques. Starting with the results of the first set of robustness checks displayed in Table 3, the PSCC-OLS quadratic model reveals that health expenditure shows an inverted U-shaped relationship with maternal mortality and life expectancy. That is, health expenditures initially worsen both outcomes but later improved them as the coefficient of the squared health expenditure is negative. However, the relationship between health expenditure and child mortality is inconclusive. The thresholds of maternal mortality and life expectancy are 2.72 and 6.67 respectively. Accounting for the level of education as the moderation variable, health expenditure has an inverted U-shaped relation with maternal mortality but a U-shaped relationship with life expectancy. The respective conditional threshold points of health expenditures on maternal mortality and life expectancy are 0.25 and 0.37, respectively. From the PSCC-FE results, the quadratic models reveal that health expenditures exert a U-shaped relationship with maternal and child mortalities. The respective threshold points are 3.58 and 4.34. On the moderating relationships, we find that a consistent U-shaped nexus between health expenditures, maternal and child mortalities. Thus, the respective conditional threshold points are 0.62 and 0.92. For the most part, these results are consistent with those of Table 2.

**Table 3:** Robustness checks (tuberculosis models)

Variables	PSCC-OLS						PSCC-FE					
	Quadratic Models			Moderation Models			Quadratic Models			Moderation Models		
	lnMAT [1]	lnCHD [2]	lnLEX [3]	lnMAT [4]	lnCHD [5]	lnLEX [6]	lnMAT [7]	lnCHD [8]	lnLEX [9]	lnMAT [10]	lnCHD [11]	lnLEX [12]
lnHEXPC	0.536*** (7.740)	− 0.0468 (− 1.265)	0.0303*** (3.553)	0.143*** (5.652)	− 0.0132 (− 0.524)	− 0.0145*** (− 3.600)	−0.209*** (− 5.918)	−0.171*** (− 3.615)	0.0262 (1.365)	− 0.113*** (− 4.414)	− 0.0982*** (− 2.945)	0.00251 (0.212)
lnEDU	0.198* (2.013)	−0.489*** (− 11.66)	0.0631*** (5.650)	2.290*** (13.92)	0.101 (0.731)	−0.114*** (− 3.476)	− 0.462*** (− 6.615)	−0.853*** (− 10.20)	0.0672*** (3.169)	− 1.320*** (− 6.762)	− 1.365*** (− 6.728)	0.0516 (0.485)
lnICT	− 0.242*** (− 8.614)	− 0.122*** (− 6.810)	0.0236*** (7.115)	− 0.285*** (− 8.962)	−0.144*** (− 7.418)	0.0327*** (7.795)	− 0.0618*** (− 8.036)	−0.0100 (− 1.055)	0.00970*** (3.428)	− 0.0492*** (− 5.848)	−0.00361 (− 0.314)	0.0118** (2.761)
lnHCON	0.166*** (5.637)	−0.311*** (− 22.59)	− 0.0657*** (− 7.280)	0.0887*** (4.196)	−0.324*** (− 22.01)	−0.0636*** (− 7.553)	−0.0572 (− 0.821)	−0.0473* (− 1.770)	0.0276** (2.100)	− 0.0708 (− 0.964)	−0.0528* (− 1.816)	0.0231** (2.189)
lnTUB	0.266*** (35.29)	0.206*** (62.44)	−0.0673*** (−20.65)	0.233*** (48.53)	0.200*** (60.18)	−0.0659*** (−20.94)	− 0.0487 (− 1.649)	0.0656*** (5.640)	−0.0605*** (− 3.699)	−0.0330 (− 0.932)	0.0751*** (5.970)	−0.0603*** (− 4.290)
lnTFP	− 0.00152 (− 0.0177)	−0.00310 (− 0.104)	−0.0146 (− 0.942)	0.0269 (0.383)	0.0119 (0.407)	− 0.0207 (− 1.313)	0.0402 (0.813)	−0.0191 (− 1.446)	−0.00830 (− 0.527)	0.0358 (0.734)	−0.0237* (− 1.929)	−0.00513 (− 0.326)
lnHEXPCSQ	− 0.0984*** (− 11.21)	−0.00820* (− 1.806)	−0.00227* (− 1.952)				0.0292*** (5.965)	0.0197*** (3.929)	−0.00323 (−1.131)			
lnHEXPC*lnEDU				−0.580*** (−19.86)	− 0.145*** (− 4.615)	0.0388*** (6.385)				0.181*** (5.290)	0.107*** (3.617)	0.00392 (0.200)
**Threshold**	**2.72**	**NA**	**6.67**	**0.25**	**NA**	**0.37**	**3.58**	**4.34**	**NA**	**0.62**	**0.92**	**NA**
Constant	0	0	0	0	0	0	0	0	0	0	0	0
Year Dummies	Yes	Yes	Yes	Yes	Yes	Yes	Yes	Yes	Yes	Yes	Yes	Yes
Observations	525	525	525	525	525	525	525	525	525	525	525	525
R-squared	0.769	0.689	0.709	0.776	0.697	0.715						
Countries	25	25	25	25	25	25	25	25	25	25	25	25
F-Statistic	6992	5364	17,567	1606	6760	1203	1282	1855	2118	56.08	204.2	364.1

Source: Authors’ Calculations

*ln* Natural logarithm, *MAT* Maternal mortality, *CHD* Child mortality, *LEX* Life expectancy at birth, *HEXPC* Health expenditures per capita, *EDUC* Education, *ICT* Internet users, *HCON* Household consumption, *TUB* People with tuberculosis, *TFP* Total factor productivity

*** *p* < 0.01, ** *p* < 0.05, * *p* < 0.1; *t*-statistics in parentheses

The results from using 5-year averages are displayed in Tables 4 and 5. Starting with Table 4 (HIV model) the results from the PSCC-OLS are similar to those displayed in Table 2. The inverted U-shaped relationship between health expenditures, maternal and child mortalities is sustained with a threshold point of 3.22 and 2.86 while the relationships with life expectancy is inconclusive though the coefficients have the expected signs. In addition, the moderating effects of education level on the nexus is sustained with inverted U-shaped relations with maternal and child mortalities and a U-shaped relation and life expectancy. The respective threshold points are 0.44, 0.46, and 0.60. From the PSCC-FE results, the quadratic effect reveals a U-shaped nexus with maternal and child mortalities and an inverted U-shaped relationship with life expectancy. The respective threshold points are 3.46, 4.27, and 4.04. Likewise, the moderation relationship depicts a U-shaped nexus with maternal mortality with the threshold point at 0.60 while the relationship between health expenditures with child mortality and life expectancy are inconclusive. These results bear semblance to those shown in Table 2.

**Table 4:** Robustness checks, 5-year averages (HIV models)

Variables	PSCC-OLS						PSCC-FE					
	Quadratic Models			Moderation Models			Quadratic Models			Moderation Models		
	lnMAT [1]	lnCHD [2]	lnLEX [3]	lnMAT [4]	LnCHD [5]	LnLEX [6]	lnMAT [7]	lnCHD [8]	lnLEX [9]	lnMAT [10]	lnCHD [11]	lnLEX [12]
lnHEXPC	0.818*** (10.34)	0.209*** (2.867)	−0.0619 (−1.225)	0.348*** (6.799)	0.152*** (11.01)	−0.0615** (−2.610)	− 0.306*** (−7.382)	− 0.164** (− 2.619)	0.0314*** (6.134)	− 0.166*** (− 10.11)	− 0.0845 (− 1.502)	−0.00744 (− 0.691)
lnEDU	0.374*** (4.325)	−0.533*** (− 8.122)	0.0910*** (4.483)	3.312*** (59.39)	0.799*** (15.06)	−0.313*** (−3.916)	− 0.541*** (− 4.865)	−0.834*** (− 8.503)	0.0546** (2.498)	−1.862*** (− 12.30)	−1.258*** (− 4.214)	−0.0415 (− 0.463)
lnHEXPCSQ	− 0.127*** (− 23.07)	−0.0366*** (− 5.108)	0.00882* (1.831)				0.0442*** (7.349)	0.0192*** (3.579)	−0.00389*** (− 5.440)			
lnHEXPC*lnEDU				−0.793*** (− 29.64)	− 0.341*** (− 44.48)	0.102*** (4.793)				0.278*** (14.18)	0.0886* (1.915)	0.0212 (1.372)
lnICT	−0.374*** (− 9.934)	− 0.197*** (−8.269)	0.0418*** (3.861)	−0.442*** (− 9.084)	−0.243*** (− 13.40)	0.0570*** (5.235)	− 0.0986*** (− 5.394)	−0.0129 (− 1.381)	0.0110*** (3.474)	− 0.0740*** (− 4.956)	−0.00774 (− 0.482)	0.0168*** (2.964)
lnHCON	0.0615 (1.155)	−0.480*** (− 10.64)	− 0.0163*** (− 2.858)	−0.0290 (− 0.411)	−0.520*** (− 9.280)	−0.00419 (− 1.001)	−0.176 (− 1.409)	−0.0576 (− 1.443)	0.0104 (0.569)	− 0.209* (− 1.715)	−0.0638 (− 1.549)	0.00173 (0.0982)
lnHIV	0.101*** (11.67)	0.131*** (13.77)	−0.0498*** (− 5.543)	0.0706*** (6.656)	0.125*** (15.19)	−0.0489*** (− 5.971)	− 0.190*** (− 7.997)	0.00868* (1.755)	0.0267 (1.564)	− 0.196*** (− 18.71)	0.0145** (2.539)	0.0152 (0.955)
lnTFP	0.0400 (0.259)	0.00291 (0.115)	− 0.0160 (− 0.833)	0.0890 (0.843)	0.0345** (2.295)	− 0.0266* (− 1.876)	0.171** (2.117)	− 0.0101 (− 0.529)	−0.0262 (− 1.555)	0.167** (2.215)	− 0.0172 (− 1.157)	−0.0185 (− 1.146)
**Threshold**	**3.22**	**2.86**	**NA**	**0.44**	**0.46**	**0.60**	**3.46**	**4.27**	**4.04**	**0.60**	**NA**	**NA**
Constant	0	0	0	0	0	0	0	0	0	0	0	0
Year Dummies	Yes	Yes	Yes	Yes	Yes	Yes	Yes	Yes	Yes	Yes	Yes	Yes
Observations	125	125	125	125	125	125	125	125	125	125	125	125
R-squared	0.652	0.583	0.651	0.685	0.614	0.692						
Countries	25	25	25	25	25	25	25	25	25	25	25	25
F-Statistic	156.8	98.33	669.8	12,423	759.1	21.28	1794	5678	1296	1827	486.1	93.16

Source: Authors’ Calculations

*ln* Natural logarithm, *MAT* Maternal mortality, *CHD* Child mortality, *LEX* Life expectancy at birth, *HEXPC* Health expenditures per capita, *EDUC* Education, *ICT* Internet users, *HCON* Household consumption, *HIV* People living with HIV, *TFP* Total factor productivity

*** *p* < 0.01, ** *p* < 0.05, * *p* < 0.1; *t*-statistics in parentheses

**Table 5:** Robustness checks, 5-year averages (TUB models)

Variables	PSCC-OLS						PSCC-FE					
	Quadratic Models			Moderation Models			Quadratic Models			Moderation Models		
	lnMAT [1]	lnCHD [2]	lnLEX [3]	lnMAT [4]	lnCHD [5]	lnLEX [6]	lnMAT [7]	lnCHD [8]	lnLEX [9]	lnMAT [10]	lnCHD [11]	lnLEX [12]
lnHEXPC	0.636*** (9.937)	−0.0184 (− 0.391)	0.0239* (2.055)	0.190*** (7.134)	0.0187 (0.377)	−0.0199*** (−3.288)	− 0.231*** (−10.44)	− 0.168** (−2.790)	0.0222*** (4.303)	− 0.110*** (− 10.72)	−0.0980** (− 2.091)	−0.00499 (− 0.955)
lnEDU	0.381** (2.781)	−0.460*** (− 9.203)	0.0566*** (4.163)	2.670*** (15.05)	0.271 (1.560)	−0.149*** (− 6.458)	− 0.543*** (− 5.914)	−0.849*** (− 8.600)	0.0690*** (4.346)	−1.340*** (− 18.71)	−1.402*** (− 5.724)	− 0.00379 (− 0.0676)
lnHEXPCSQ	− 0.111*** (− 11.00)	− 0.0112** (− 2.315)	− 0.00134 (− 1.234)				0.0319*** (12.06)	0.0202*** (3.708)	−0.00259*** (− 2.946)			
lnHEXPC*lnEDU				−0.639*** (− 24.81)	− 0.179*** (− 3.566)	0.0455*** (12.24)				0.166*** (8.657)	0.116*** (3.317)	0.0159* (1.751)
lnICT	−0.316*** (− 6.610)	− 0.149*** (− 36.03)	0.0262*** (6.725)	−0.367*** (− 7.564)	−0.182*** (− 20.18)	0.0384*** (10.30)	− 0.0881*** (− 5.788)	−0.0175 (− 1.492)	0.0134*** (3.167)	− 0.0776*** (− 4.717)	−0.00892 (− 0.539)	0.0181*** (3.307)
lnHCON	0.169*** (5.751)	−0.335*** (− 17.43)	− 0.0721*** (− 7.633)	0.0628*** (4.357)	−0.356*** (− 37.73)	−0.0686*** (− 9.477)	−0.251* (− 1.726)	−0.0909*** (− 3.326)	0.0546* (1.762)	− 0.270* (− 1.797)	−0.108*** (− 5.449)	0.0424 (1.617)
lnTUB	0.261*** (25.54)	0.205*** (44.49)	−0.0645*** (− 11.10)	0.227*** (54.45)	0.197*** (47.27)	−0.0627*** (− 11.99)	− 0.0344 (− 0.867)	0.0805*** (5.567)	−0.0677*** (− 3.245)	−0.0189 (− 0.364)	0.0917*** (5.949)	−0.0652*** (− 3.544)
lnTFP	0.105 (0.596)	0.0314 (0.8 81)	−0.0211 (− 1.005)	0.135 (0.975)	0.0492 (1.530)	−0.0277 (− 1.380)	0.158* (1.829)	− 0.00702 (− 0.489)	−0.0266 (− 1.027)	0.149* (1.740)	− 0.0107 (− 1.479)	−0.0210 (− 0.845)
**Threshold**	**2.86**	**NA**	**NA**	**0.30**	**NA**	**0.44**	**3.62**	**4.16**	**4.29**	**0.66**	**0.84**	**NA**
Constant	0	0	0	0	0	0	0	0	0	0	0	0
Year Dummies	Yes	Yes	Yes	Yes	Yes	Yes	Yes	Yes	Yes	Yes	Yes	Yes
Observations	125	125	125	125	125	125	125	125	125	125	125	125
R-squared	0.769	0.687	0.721	0.777	0.698	0.731						
Countries	25	25	25	25	25	25	25	25	25	25	25	25
F-Statistic	2492	7761	1472	4543	2208	3791	1595	1179	12,295	1466	136.7	4260

Source: Authors’ Calculations

*ln* Natural logarithm, *MAT* Maternal mortality, *CHD* Child mortality, *LEX* Life expectancy at birth, *HEXPC* Health expenditures per capita, *EDUC* Education, *ICT* Internet users, *HCON* Household consumption, *TUB* People with tuberculosis, *TFP* Total factor productivity

*** *p* < 0.01, ** *p* < 0.05, * *p* < 0.1; *t*-statistics in parentheses

We further substituted HIV with those living with tuberculosis (TUB) and the results are displayed in Table 5. From the PSCC-OLS results, we find that the quadratic relationship depicts an inverted U-shaped relation with a threshold point at 2.86 while the relationships with child mortality and life expectancy are inconclusive. On the moderating relationships, we find an inverted U-shaped and a U-shaped nexus with maternal mortality and life expectancy while that of child mortality is inconclusive. The threshold points are 0.30 and 0.44, respectively. From the PSCC-FE results, a U-shaped relation with maternal and child mortalities and an inverted U-shaped nexus with life expectancy. The respective threshold points are 3.62, 4.16, and 4.29. From the moderation models, a U-shaped nexus exists between health expenditures and maternal and child mortalities with the respective threshold points at 0.66 and 0.84. Again, these results somewhat sustain those in Table 3. Overall, we submit that the relationship between health expenditures and the three health outcomes is nonlinear.

### Applicability and results implication

This discusses the applicability of the results for SSA countries. Table 6 presents the mean of public health expenditure vis-à-vis its thresholds. We show that almost, all the countries in SSA have not met the computed threshold of 3.65 public health expenditure, except, Lesotho and Namibia. Although, the computed threshold of 3.65% of GDP (Gross Domestic Product) relates to life expectancy at birth, it is taken as the highest bench mark of threshold that must be achieved by each country since all the health outcomes are achieved simultaneously. This is because in the process of achieving life expectancy at birth, all other health outcomes like maternal mortality and infant mortality would have been achieved. Therefore, every country must aspire to allocate at least 3.65% of their GDP to health for an all-encompassing health performance. It is therefore evident that public health financing is underfunded among all the countries in SSA and there is a need for urgent campaign towards increase in public health expenditure in SSA.

**Table 6:** Mean of public health expenditure and thresholds

Country	Public health expenditure Mean	Threshold of Public health expenditure (Highest range)	Remarks
Angola	3.4884	3.65	Below Threshold
Burkina Faso	1.6466	3.65	Below Threshold
Benin	0.7083	3.65	Below Threshold
Botswana	3.5556	3.65	Below Threshold
Burundi	2.0699	3.65	Below Threshold
Cameroon	0.7313	3.65	Below Threshold
Central Africa Republic	1.2228	3.65	Below Threshold
Cote D’Ivoire	0.8921	3.65	Below Threshold
Eswatini	3.3083	3.65	Below Threshold
Gabon	1.4304	3.65	Below Threshold
Kenya	1.6968	3.65	Below Threshold
Lesotho	4.2560	3.65	Threshold met
Mauritania	1.1607	3.65	Below Threshold
Mauritius	1.8724	3.65	Below Threshold
Mozambique	1.9735	3.65	Below Threshold
Namibia	4.3361	3.65	Threshold met
Niger	1.8266	3.65	Below Threshold
Nigeria	0.6757	3.65	Below Threshold
Rwanda	1.9087	3.65	Below Threshold
Sierra Leone	1.5402	3.65	Below Threshold
South Africa	3.4884	3.65	Below Threshold
Senegal	1.3125	3.65	Below Threshold
Sudan	1.5549	3.65	Below Threshold
Tanzania	1.5488	3.65	Below Threshold
Togo	0.9728	3.65	Below Threshold

Source: Authors’

## Conclusion and policy implications

The study concludes that public health expenditure and educational quality are significant in driving changes in maternal mortality, child mortality and life expectancy at birth in SSA. The quadratic results support the existence of a non-linear relationship between health expenditure and the health outcome indices. Therefore, the quadratic results, reveal the sustainable public health expenditure thresholds that can achieve better health outcomes as 3.16; for maternal mortality, 2.73, for child mortality and 3.65 for life expectancy. The results of the interactive effect of health expenditure and educational quality on health outcomes reflects that an inverted U-shaped curve exists between health expenditures, educational quality and the mortality models while a U-shaped curve hold for life expectancy model. Since the shape for the parabola for the mortality models is an inverted U-shape suggesting that beyond 23.57 and 15.33, health expenditures per capita will contribute significantly to reducing both maternal and child mortality. In the same vein, following the U-shape relationship, it follows that beyond the threshold point of 38.47, health expenditures per capita will contribute significantly to improving life expectancy. The conditional result reflects the point at which educational quality *negatively* influences the impact of health expenditures on maternal and child mortality as 1.77 and 2.61 respectively. The moderation results reveal, the educational threshold points of 1.51 and 1.49 induce a drop in maternal and child mortalities while a point beyond 1.84 exerts an improvement in life expectancy.

The policy outcomes are not far-fetched. First, the government and stakeholders should embrace strategies that will enhance public health expenditure and the education quality because an improvement in these variables will bring about a meaningful impact on the health outcomes (maternal mortality, child mortality and life expectancy at birth). Secondly, there is the need to not just increase public health expenditure but meet the minimum level of sustainable thresholds estimated in this study being the bench mark that will enable the SSA to operate within the Universal health Coverage standard because most of the SSA countries are operating below the sustainable thresholds of public health expenditure. Third, education should be considered as a complementary health variable, given the modifying effect of education and health expenditure on health outcomes, therefore, this study supports the Grossman theory of health. Finally, we recommend that government and stake holders in the health sector should strive to meet the minimum thresholds of educational quality that will enhance public health expenditure for better health outcomes in SSA. Overall, our results show that the effect of health expenditures on each health outcome is dependent on the strength of educational quality, and therefore, educational quality should be considered as an important requirement in modelling any health framework. In addition, a knowledge of the threshold effect of public health expenditure on health outcome will help to curb and minimize, corruption and wastages in the health sector because the government will know the appropriate percentage of the GNP that must go to health and education for better health outcomes. Therefore, the excesses and shortages in health financing will be reduced, ceteris paribus. For future work and subject to data availability, control variables such as GDP per capita, urbanization, development aid etc. which could change the signs and statistical significance of the coefficients used to calculate the thresholds may be considered.

## Data Availability

The data adopted in the study are available in public domains and can be provided on request.

## Abbreviations

AIDS: Acquired Immune Deficiency Syndrome
ASEAN: Association of South East Asian Nations
CH D: Child Mortality
CSD: Cross Sectional Dependence
EDU: Education
GDP: Gross Domestic Product
GMM: Generalized Method of Moment
ICT: Information Communication Technology
LEB: Life Expectancy at Birth
MDG: Millennium Development Goals
MMR: Maternal Mortality Rate
NEET: Not in Employment, Education or Training
OECD: Organization for Economic Co-operation and Development
OLS: Ordinary Least Square
PARDL: Panel Auto Regressive Distributed Lag
PWT: Penn World Table
PHE: Public Health Expenditure
PSCC: Panel Spatial Correlation Consistent
PSCC-OLS: Panel Spatial Correlation Consistent- Ordinary Least Square
PSCC-FE: Panel Spatial Correlation Consistent-Fixed Effect
SDG: Sustainable Development Goals
SAARC: South Asian Association for Regional Cooperation
SSA: Sub-Saharan Africa
TFP: Total Factor Productivity
TB: Tuberculosis
UNDP: United Development Program
WDI: World Development Indicators
WLS: Weighted Least Square
W.H.O: World Health Organization

## Footnotes

1 Weighted least squares

2 Angola, Burkina Faso, Benin Republic, Botswana, Burundi, Cameroon, Central African Republic, Cote D’Ivoire, Eswatini, Gambia, Kenya, Lesotho, Mauritania, Mauritius, Mozambique, Namibia, Niger, Nigeria, Rwanda, Sierra Leone, South Africa, Senegal, Sudan, Tanzania, and Togo.

3 The level and moderation relationships are positive but statistically not significant. Hence, the threshold point cannot be computed.
